# Immune Modulation as a Therapeutic Option During the SARS-CoV-2 Outbreak: The Case for Antimalarial Aminoquinolines

**DOI:** 10.3389/fimmu.2020.02159

**Published:** 2020-08-28

**Authors:** Joana Vitte, Moïse Michel, Soraya Mezouar, Aïssatou Bailo Diallo, Asma Boumaza, Jean-Louis Mege, Benoit Desnues

**Affiliations:** ^1^Aix Marseille Univ, IRD, APHM, MEPHI, Marseille, France; ^2^IHU-Méditerranée Infection, Marseille, France

**Keywords:** COVID-19, SARS-CoV-2, chloroquine, hydroxychloroquine, immune modulation

## Abstract

The rapid spread, severity, and lack of specific treatment for COVID-19 resulted in hasty drug repurposing. Conceptually, trials of antivirals were well-accepted, but twentieth century antimalarials sparked an impassioned global debate. Notwithstanding, antiviral and immunomodulatory effects of aminoquinolines have been investigated *in vitro, in vivo* and in clinical trials for more than 30 years. We review the mechanisms of action of (hydroxy)chloroquine on immune cells and networks and discuss promises and pitfalls in the fight against SARS-CoV-2, the agent of the COVID-19 outbreak.

## Introduction

The rapid spread and severity of the coronavirus disease 2019 (COVID-19) have been accentuated by the lack of a specific treatment and resulted in hasty drug repurposing. Conceptually, trials of molecules like remdesivir or lopinavir already used for combatting emergent viruses were well-accepted. Conversely, repurposing drugs that collective memory associated with twentieth century antimalarial treatment sparked an impassioned global debate. Notwithstanding, antiviral and immunomodulatory effects of chloroquine (CQ) and hydroxychloroquine (HCQ), now including Severe Acute Respiratory Syndrome Coronavirus 2 (SARS-CoV-2), have been investigated for more than 30 years *in vitro, in vivo* and in clinical trials (1–3). Among 2,654 clinical trials on COVID-19 registered with Clinicaltrials.gov from 43 countries and all continents by mid-July 2020, 239 included HCQ treatment or prophylaxis while 82 addressed CQ (clinicaltrials.gov, accessed July 17, 2020). At the same date, published available results were scarce, with only 13 papers identified as clinical trials in a PubMed search with the filters HCQ and 1 year, and 20 for CQ (pubmed.ncbi.nlm.nih.gov, accessed July 17, 2020). Here, we will review and comment CQ/HCQ effects on immune responses, aiming to identify their promises and pitfalls in the fight against SARS-CoV-2, the agent of the COVID-19 outbreak.

## HCQ and CQ: Basic Knowledge of Structure and Chemistry

**Figure 1:**
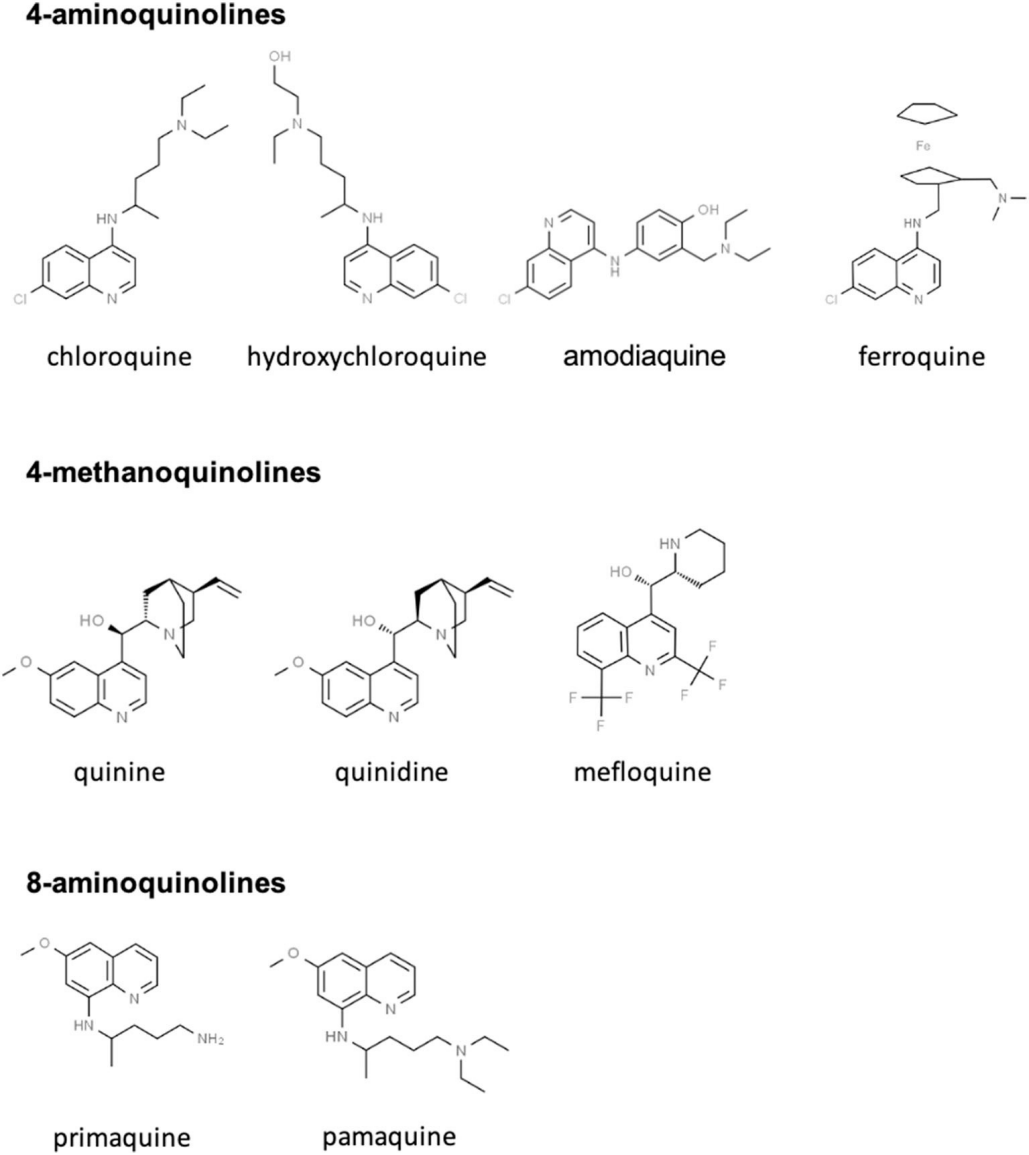
Skeletal structures of some quinoline-based antimalarial drugs. Structures were taken from https://www.chemspider.com/ (with written permission).

CQ and HCQ belong to the chemical class of quinolines, which has been intensively investigated for the development of potent antimalarial agents. Antimalarial quinoline derivatives belong to three main categories: the 4-aminoquinolines, which include CQ, HCQ, amodiaquine, and ferroquine, the 4-methanoquinolines, represented by quinine, quinidine, and mefloquine, and the 8-aminoquinolines, comprising primaquine and pamaquine, the latter also known as plasmoquine (Figure 1). CQ-resistance was soon evolved by *Plasmodium falciparum*, resulting in discontinued therapeutic and prophylactic use (4). Yet, it was observed during World War II that soldiers receiving CQ antimalarial prophylaxis experienced anti-inflammatory effects and attenuated arthritis symptoms. Currently, CQ and HCQ have been cleared by the US Food and Drug Administration (FDA) for rheumatoid arthritis (RA) and systemic lupus erythematosus (SLE), while clinical research trials are in progress for Sjögren's syndrome, graft-vs.-host disease, cancer, and nanomedicine approaches (5–9). CQ and HCQ also demonstrated antiviral (10–12), antibacterial (13–15), antifungal (16), and immunomodulatory (17, 18) properties, resulting from distinct, yet not fully characterized mechanisms (16). Aminoquinolines are not an isolated example of antimalarials repurposed for emerging and re-emerging viral infections. Artemisinin derivatives, methanoquinolines, and antimicrobial drugs are further examples (Table 1).

**Table 1:** Antimalarial drugs with antiviral actions.

	**Methanoquinolines**	**Aminoquinolines**	**Artemisinin derivates**	**Antibiotics**		
**Name**	**Quinine/quinidine, mefloquine**	**Chloroquine, hydroxychloroquine, amodiaquine, ferroquine, primaquine, pamaquine**	**Artemisinin, dihydroartemisinin, artesunate, artemether**	**Doxycycline**	**Sulfonamides**	**Atovaquone**
Origin	Quinine/quinidine: alkaloid enantiomers from *Chinchona officinalis* Mefloquine: synthetic derivation of quinine	Synthetic derivation of quinine	Artemisinin: sesquiterpene lactone from *Artemisia annua* Dihydroartemisinin, artesunate and artemether: synthetic derivation of artemisinin	Synthetic derivation of oxytetracycline	Synthetic derivation of sulfonic acid	Synthetic naphtoquinone
Discovery/1st report	Seventeenth century (quinine/quinidine), 1974 (mefloquine)	1934 (chloroquine), 1949 (hydroxychloroquine), 1948 (amodiaquine), 1994 (ferroquine), 1946 (primaquine), 1926 (pamaquine)	1972 (artemisinin), 1986 (dihyroartemisinin), 1987 (artesunate and artemether)	1957	1935	1991
Anti-malarial mode of action	Inhibition of parasite heme polymerase, preventing the conversion of heme to hemazoin		Binding and cleavage of intraparasitic heme-iron to produce toxic free radicals	Inhibition of apicoplast protein synthesis	Inhibition of dihydropteroate synthetase and dihydrofolate reductase	Inhibition of mitochondrial electron transport (cytochrome *bc1* complex)
Anti-viral activity (*in vitro*, animal models, humans)	Influenza virus, HSV-1, DENV, JCPyV, ZIKV	CHIKV, ZIKV, DENV, EBOV, coronaviruses (SARS-CoV, MERS-CoV, HCoV-229E and HCoV-OC43), HCV, HIV-1 and−2, influenza viruses (H1N1 and H3N2), enteroviruses, SFTSV, NDV, HSV-1	HCMV, HBV, HCV, HPV, CPV, HIV, HHV-6, EBV, BKPyV, JCPyV, EBOV	DENV, CHIKV, VSV, influenza A virus	HIV-1 (in combination with antiretroviral therapy), KHSV	CHIKV, ZIKV

*BKPyV, BK polyomavirus; CHIKV, Chikungunya virus; CPV, canine parvovirus; DENV, dengue virus; EBOV, Ebola virus; EBV, Epstein-Barr virus; HBV, hepatitis B virus; HCMV, Human cytomegalovirus; HCoV, human coronavirus; HCV, hepatitis C virus; HHV, human herpesvirus; HIV, Human Immunodeficiency Virus; HPV, human papillomavirus; HSV, Herpes simplex Virus; JCPyV, human polyomavirus 2; KHSV, Kaposi's sarcoma-associated herpesvirus (HHV-8); MERS-CoV, Middle East Respiratory Syndrome coronavirus; NDV, Newcastle Disease Virus; SARS-CoV, Severe Acute Respiratory Syndrome Coronavirus; SFTSV, Severe Fever with Thrombocytopenia Syndrome Bunyavirus; VSV, Vesicular stomatitis virus; ZIKV, Zika virus*.

CQ/HCQ are amphiphilic, diprotic weak bases (pK_a1_ = 8.1, pK_a2_ = 10.2 and pK_a1_ = 8.3, pK_a2_ = 9.7 at 37°C, respectively). As conjugate bases, CQ/HCQ freely cross plasma and organelle membranes, then accumulate in acidic intracellular compartments, e.g., microsomes, endosomes, and lysosomes, where they act as proton acceptors (4, 19). Consequently, these lysosomotropic agents interfere with organelle and lysosome acidification. Lysosomes are intracellular compartments hosting vital processes such as lipid metabolism, energy production, intracellular trafficking, and cell signaling (20). The latter requires the assembly of critical protein complexes, e.g., molecular target of rapamycin complex 1 (mTORC1) on lysosomal membranes and the regulation of the lysosomal store of Ca^2+^ which can be mobilized upon stimulation. Hence, interference with endosome and lysosome acidification in turn inhibits essential cell functions, e.g., proteolysis, chemotaxis, phagocytosis, phagosome conversion, and antigen presentation (21, 22). Importantly, CQ and HCQ similarly affect microorganismal enzymes and receptors, including virus-diverted cellular processes (8).

## General Mechanisms of CQ/HCQ Actions

CQ/HCQ affect multiple cellular processes irrespective of cell specialization (Table 2).

**Table 2:** Main mechanisms of chloroquine and hydroxychloroquine action at the cellular level.

**Cellular compartment**	**Target**	**CQ/HCQ action**	**Cellular consequence**	**Pathophysiological implications**
Plasma membrane	• PICALM/CALM	• Downregulation of PICALM/CALM	• Plasma membrane stabilization • Inhibition of clathrin-cargo fusion • Defective endosome formation	• Decreased clathrin-dependent and independent endocytosis • Prevention of endosome-lysosome fusion • Endosomal stasis • Impaired plasma membrane receptor recycling
	• Clathrin	• Decreased clathrin expression at the plasma membrane		
	• Bitter taste receptors (TAS2R)	• Agonist	• Inducible defense mechanisms	• Increased NO production • Increased mucociliary clearance • Anti-inflammatory effects • Muscle relaxation
Cytoplasm	• MAP kinase, NF-kB	• Inhibition	• Impairs MAPK activation and NF-kB translocation	• Downregulation of the proinflammatory cascade leading to TNFα and IL-6
	• Phospholipase A2	• Inhibition	• Inhibition of phospholipid mediator release	• Anti-inflammatory, anti-platelet aggregate
Intracellular organelles	• Acidic intracellular compartments	• Proton capture	• pH increases in endosomes, lysosomes, Golgi vesicles, microsomes	• Impairment of protease activity in the affected organelles (cellular and viral proteases, post-translational editing) • Impairment of endosomal pattern recognition receptors, e.g., endosomal toll-like receptors • Impaired antigen presentation • decreased intracellular trafficking to lysosomes • Autophagy inhibition
	• Inositol-1,4,5-trisphosphate receptor on the membranes of intracellular Ca^2+^ stores	• Inhibition of IP3 binding to its receptor	• Antagonism of intracellular Ca^2+^ mobilization mechanisms	• Further impairment of efficient endocytosis of the ligand-receptor complexes • Antagonism of cellular activation • Activation of the TFEB signaling pathway
Nucleus	• DNA	• DNA intercalation	• Replication impairment (at high CQ concentrations around 1 mM)	• Anti-infectious activity
*Plasmodium*	• Phagocytic vacuole	• β-hematin binding	• Impaired heme detoxification, accumulation of free hemin	• Antimalarial activity

*CQ, chloroquine; DNA, deoxyribonucleic acid; IL, interleukin; IP3, inositol-trisphoshpate; MAP kinase, Mitogen-Activated Protein kinase; NF-kB, Nuclear Factor kappa-light-chain-enhancer of activated B cells; NO, nitric oxide; PICALM/CALM, phosphatidylinositol binding clathrin assembly lymphoid myeloid leukemia protein; TAS2R, bitter taste receptors; TFEB, transcription factor specific for E-Box sequences; TNFα, Tumor Necrosis Factor α*.

CQ/HCQ impair endosome-lysosome fusion through proton capture and pH increase, resulting in decreased clathrin expression at the plasma membrane, an endosomal stasis sometimes compared to a traffic jam upstream the lysosomal compartment, an increase in the number of intracellular vacuoles and a general decrease in endocytic trafficking and membrane receptor recycling (8, 23, 24). Moreover, CQ/HCQ specifically downregulate plasma membrane expression of PICALM/CALM (phosphatidylinositol binding clathrin assembly lymphoid myeloid leukemia protein), a transmembrane protein involved in clathrin-mediated and probably also clathrin-independent endocytosis (8). PICALM acts as a membrane curvature sensor and clathrin-cargo adaptor (25, 26). PICALM downregulation provides the mechanistic explanation for membrane stabilization with CQ/HCQ (26).

Proton capture explains CQ/HCQ-induced pH increase and organelle deacidification, affecting any subcellular compartment with baseline pH lower than the surrounding cytoplasm, e.g., lysosomes, Golgi vesicles, and microsomes. CQ/HCQ thus interfere with local proteases and endosomal pattern recognition receptors (PRRs), notably toll-like receptors (TLRs), which sense viral nucleic acids (15, 19, 27, 28). Impaired endosomal/lysosomal protease activity disrupts cellular and infectious processes. Increased intraorganellar pH is a general mechanism hampering the intracellular development of microorganisms requiring an acidic milieu: viruses, bacteria (e.g., *Coxiella burnetii*, the causal agent of Q fever, and *Tropheryma whipplei*, the bacterium responsible of Whipple's disease), or fungi (e.g., *Candida* sp.) (14, 16).

Disruption of the acidic environment of intracellular vesicular compartments results in abnormal proteolytic processing and thus alters the post-translational steps of viral, but also cellular, protein biogenesis. This provides the first mechanistic explanation of the interference of CQ and analogs with viral replication and spread, and with host receptor, cytokine, and chemokine production. As an example, raised intravesicular pH interferes with the activity of glycosyltransferases, leading to abnormal glycosylation of viral envelope glycoproteins such as SARS-CoV spike (29) and Human Immunodeficiency virus (HIV)-1 gp120 (30, 31). Virions carrying abnormally glycosylated envelope proteins are less infective and less prone to induce a strong cytopathogenic effect (4, 30, 31). Impaired glycosyl transferase activity also affects terminal glycosylation of hACE2 (human angiotensin-converting enzyme 2) (29), a SARS-CoV and SARS-CoV-2 receptor (32, 33). CQ impairs terminal N-glycosylation, but not core glycosylation, of hACE2, which takes place in the Golgi complex, and does not alter the amount of hACE2 protein expressed by Vero-6 cells (29).

CQ/HCQ interfere with the activation of at least two signaling pathways: Mitogen-Activated Protein kinases (MAPK) and Nuclear Factor kappa-light-chain-enhancer of activated B cells (NF-κB), affecting metabolic and proinflammatory responses (34–41). Through Ca^2+^ release from intracellular stores, CQ/HCQ also interfere with downstream activation of TFEB (transcription factor specific for E-Box sequences), a major enhancer of gene transcription for lysosomal biogenesis, lysosomal function, and metabolic resetting of macrophages toward glycolysis, an energetic pathway associated with increased activity (42, 43).

Inhibition of lysosomal trafficking affects upstream (endocytosis) but also downstream (autophagy) processes. Autophagy inhibition is currently fueling sustained interest in CQ/HCQ as chemosensitizers for cancer therapy (7, 9, 22, 44–46). However, autophagy inhibition also modulates dendritic cell (DC) and macrophage activation and polarization (47, 48). At the organ level, CQ/HCQ-induced autophagy inhibition has been reported as either protective or aggravating in cardiovascular (49, 50), pulmonary (51, 52), renal, and hepatic injuries (see below). Finally, autophagy inhibition interferes with viral replication, which needs cell constituent recycling through autophagy.

CQ is a broad-spectrum agonist of bitter taste receptors (TAS2R), a family of G-protein receptors expressed in lungs (airway smooth muscle, airway epithelial cells, lung macrophages, mast cells) and leukocytes, notably lymphocytes, innate lymphoid cells, and monocytes. TAS2R stimulation induces defense mechanisms such as an increase in NO (nitric oxide) production, stimulating mucociliary clearance and exerting antibacterial, anti-inflammatory and muscle relaxant effects (53–55).

Other experimentally identified CQ/HCQ targets include phospholipase A2 (56–59), porphyrins (60, 61), and even DNA at high CQ concentrations around 1 mM (42) (Table 2).

Taken together, data summarized in this section emphasize the complexity of CQ/HCQ effects. Far from mere intravacuolar proton accepters, CQ/HCQ exert specific actions such as interference with Ca^2+^ intracellular signaling, plasma membrane stabilization, and direct binding to plasma membrane receptors. Some of these mechanisms have been discovered in recent years, underscoring the potential for repurposed molecules.

## Mechanisms of CQ/HCQ-Induced Immune Modulation

Reports from the field of autoimmune diseases, mainly SLE, RA, and antiphospholipid syndrome (APS), have accounted for most of the twentieth century knowledge on immunomodulatory effects of CQ/HCQ. During SLE and primary APS, HCQ administration decreases levels of type I IFN in patients' serum, and expression of type I interferon (IFN)-inducible genes in peripheral blood mononuclear cells (62). In a retrospective cohort of 3,679 Spanish SLE patients, HCQ treatment conferred an odds ratio of 0.5 for polyautoimmunity (63). Healthcare registry data for 220 million individuals showed that HCQ intake was associated with a lower risk for coronary artery disease (64). This effect was replicated *in vitro* as HCQ-induced attenuation of human aortic endothelial cell activation upon exposure to proinflammatory cytokines (64). Overall, HCQ is considered as a safe and effective treatment for SLE, lowering the risk for clinical flares and conversely leaving patients at risk shortly after its discontinuation (18, 65). Outside the field of autoimmune diseases, CQ/HCQ-induced immunomodulatory effects in humans have been reported as beneficial in idiopathic interstitial pneumonia (66–68) and diabetes mellitus (69), among other conditions.

Reports of CQ/HCQ-induced immunomodulation in various diseases are associated to a broad range of actions on multiple immune cell types, and CQ/HCQ effects on measurable endpoints of the immune response are well-documented. Examples are given below.

### Monocytes, Macrophages and Dendritic Cells

The monocyte-macrophage system is an important target of CQ/HCQ, especially as it orchestrates downstream modulation of T cell populations (7, 43).

**Figure 2:**
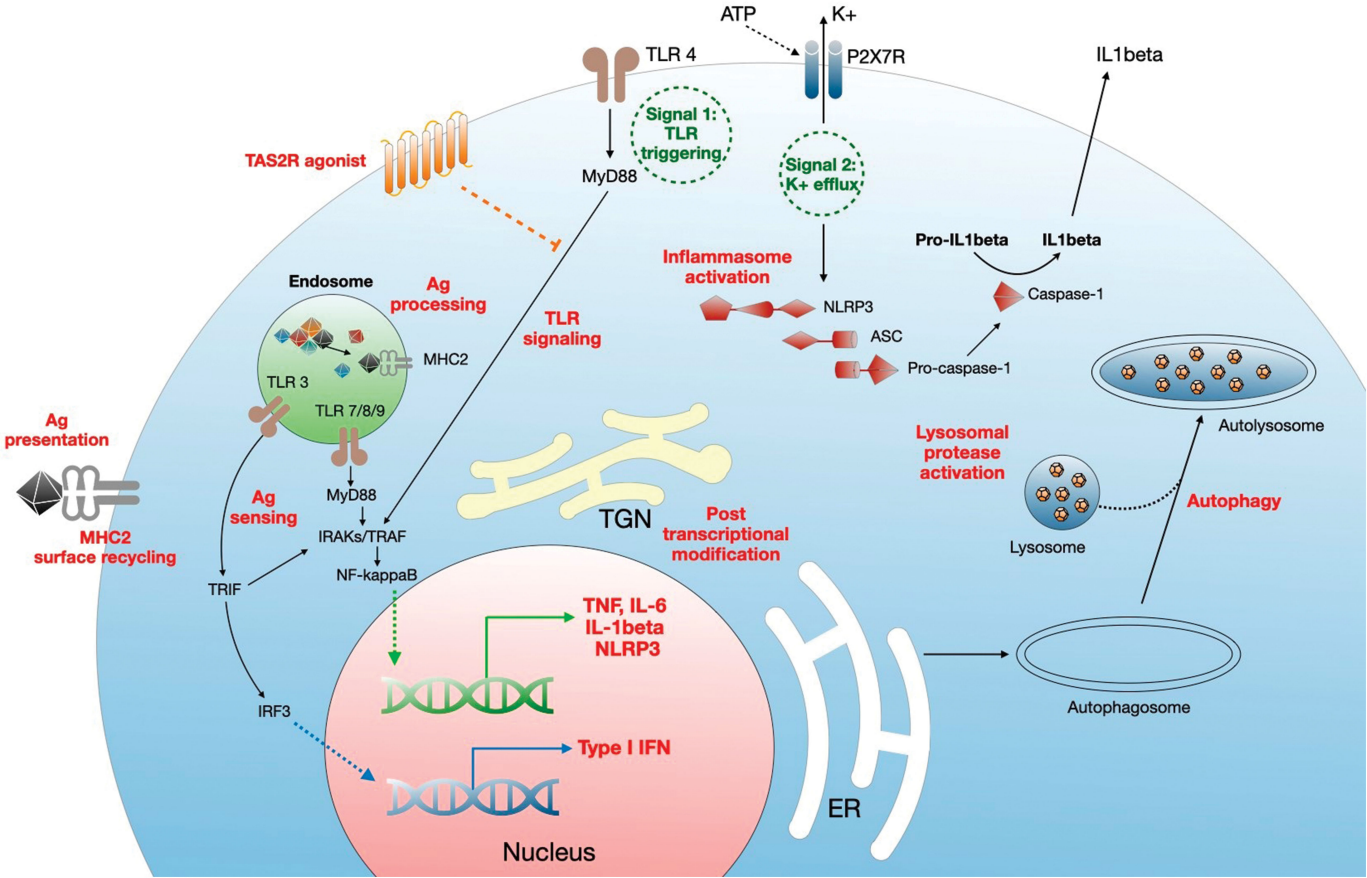
Mechanisms of regulation of monocyte/macrophage functions by CQ/HCQ. CQ/HCQ regulate macrophage responses at different levels. First, they increase the pH of intracellular organelles such as endosomes, endoplasmic reticulum (RE), Golgi apparatus and trans Golgi network (TGN), and lysosomes. This results in inhibition of fusion between nascent autophagosome and lysosomes resulting in autophagy inhibition. Deacidification of TGN affects posttranscriptional modifications such as glycosylation, resulting in protein dysfunction. CQ/HCQ also increase the pH of endosomes, leading to alteration of antigen processing, alteration of nucleic acid sensing by endosomal TLRs (which results in decreased MyD88- and TRIF-dependent responses) and impaired antigen presentation through defective antigen (Ag) loading on MHC2 molecules, which is further diminished by reduced cell surface recycling of Ag-MHC2 complexes. CQ/HCQ affect inflammasome assembly and activation by inhibiting both signal 1 (TLR activation) and signal 2 (K^+^ efflux), through decreased activity of lysosomal proteases. Finally, CQ/HCQ act as a broad direct agonist for bitter taste receptors (TAS2R) at the plasma membrane, resulting in inhibition of LPS-mediated cell activation (see text for details).

CQ/HCQ modulate the monocyte-macrophage axis through multiple pathways (Figure 2), including deacidification of intracellular organelles and disruption of cytokine production. Endosomal pH increase disrupts nucleic acid binding to endosomal TLRs, such as TLR-3, TLR-7, and TLR-9, thereby inhibiting endosomal TLR-mediated induction of type I IFN (19, 27, 28). In addition, one of the most prominent targets of CQ/HCQ during infection is the production of TNFα (Tumor Necrosis Factor α), a major proinflammatory cytokine driving a positive feedback loop of type 1 macrophage activation, oxygen and nitrogen reactive species generation, further proinflammatory mediators release, and more efficient phagocytosis (70). Lipopolysaccharide (LPS) signaling through TLR-4 is a potent inducer of TNFα release from macrophages. CQ blocks the conversion of membrane-bound TNFα to the soluble form and decreases the stability of interleukin (IL)-6 and IL-1β mRNA, thus impairing TNFα, IL-6, and IL-1β release from LPS-stimulated macrophages (36). CQ also inhibits the NLRP3 (Nod-Like Receptor Family Pyrin domain containing 3) inflammasome in LPS-treated macrophages, targeting both the priming and the assembly signals. CQ treatment of murine and human bone marrow-derived macrophages impaired NF-κB and MAPK activation and inhibited the priming signal of NLRP3 activation, resulting in the decrease of LPS-induced IL-1β, IL-18, and NLRP3. CQ also inhibited the second signal for NLRP3 inflammasome activation, i.e., inflammasome assembly, as shown by the lack of caspase-1 activation and ASC (apoptosis associated speck-like protein containing a CARD) specks formation (71). This may relate to altered processing of lysosomal cysteine proteases such as cathepsins B, C, L, S, and Z, which are involved in NLRP3 activation (72). *In vivo*, CQ administration in a mouse model of endotoxic shock was associated with decreased systemic, lung, and peritoneal levels of proinflammatory cytokines IL-1β and IL-18, lower pulmonary levels of NLRP3 and caspase-1 and improved survival (73). Similarly, HCQ inhibition of cathepsins B and L blunted NLRP3 inflammasome activation in a mouse model of renal ischemia-reperfusion injury and an *in vitro* model using human renal proximal tubule cells HK-2 (41).

Deacidification of the endosomes impairs activation of conventional (myeloid, mDCs) and plasmacytoid (pDCs) subsets of DCs. Decreased efficiency of endosomal TLR signaling interferes with pDCs activation, while activation of mDCs is impaired through a decrease in interleukin-1 receptor associated kinase 4 (IRAK-4) and IFN regulatory factor-7 (IRF-7) downstream MyD88 (Myeloid Differentiation primary response 88) signaling, and through downregulation of IFNα synthesis, collectively resulting in the downregulation of virus-induced immune activation (74).

Deacidification of intracellular organelles is not the only immunomodulatory mechanism of CQ. Extragustatory TAS2R stimulation exerts anti-inflammatory properties and bronchodilator activities, and CQ is a TAS2R agonist (53–55). TAS2R stimulation in lung macrophages exposed to CQ significantly inhibited LPS-mediated production of TNFα, CCL3 (C-C motif chemokine ligand 3) and CXCL8 (C-X-C motif chemokine ligand 8), but also of the regulatory cytokine IL-10, suggesting that, at least in this macrophage population, inhibition of the proinflammatory cytokines was not related to IL-10 overexpression (55).

However, CQ effects may revert from protection to aggravation of the inflammatory process. For example, in the context of sterile inflammatory conditions or viral-induced cytokine storm, when macrophages receive proinflammatory signals such as exogenous IL-1β, CQ inhibits the turn-over of plasma membrane IL-1 receptor, thus supporting persistent macrophage activation (47).

CQ/HCQ are able to re-tune tumor-associated macrophages from a permissive M2 to an antitumor M1 phenotype, resulting in decreased immunosuppressive cell infiltration, and enhanced antitumor T-cell immunity (7, 43). CQ treatment induced Ca^2+^ release from lysosomes, which in turn activated NF-κB, but also TFEB (7, 43). To our knowledge, the apparent contradiction between the classical view of CQ-induced inhibition of the M1 phenotype and experiments showing macrophage reprogramming from M2 to M1 has not been addressed yet. We speculate that the precise tuning of intracellular signaling depends on macrophage microenvironment, polarization, and activation status at the initiation of the CQ/HCQ treatment, together with a possible modulation of cytokine actions on target cells, given that lysosome deacidification may impair cytotoxic effects, but also potentiate them, e.g., through autophagy-related inhibition of apoptosis.

### Mast Cells (MCs) and Granulocytes

Few studies have addressed the effects of CQ/HCQ on other innate immune cells. Nevertheless, it was reported that CQ inhibits mast cell activation after TAS2R engagement, through interference with membrane phospholipid turnover, as demonstrated *in vitro* by reduced histamine and prostaglandin release (75, 76). These observations were confirmed *ex vivo* in human primary MCs (38). MC cytoplasm is notoriously packed with secretory granules, which display an acidic pH of 5.2 to 6.1, optimal for MC proteases (77). Experiments performed with bafilomycin A1, an inhibitor of vacuolar-type H^+^-ATPase (V-ATPase) sharing with CQ/HCQ the lysosomal tropism and the ability to increase pH in acidic compartments, showed that disruption of the acidic pH of MC secretory granules induced tryptase degradation (77). CQ/HCQ would be expected to reproduce such findings, whose main interest resides in the abrogation of the proinflammatory signals and effectors that are conveyed by intact MC proteases, such as tryptase, the best known in humans, which is involved in chronic allergic inflammation and tissue remodeling (77–79).

CQ acting *via* a TAS2R-dependent mechanism was shown to exert further anti-inflammatory effects. For example, CQ-induced TAS2R signaling in a mouse model of allergic asthma inhibited neutrophil and eosinophil chemotaxis, reducing disease severity (54).

Autophagy contributes to the production of neutrophil extracellular traps (NETs), which may become self-harming during COVID-19, sepsis, sterile inflammation, or thrombogenic conditions (80, 81). CQ inhibition of neutrophil autophagy has a reported beneficial effect on NETs formation and on the subsequent local and systemic inflammation (82). AMP (5′ adenosine monophosphate-activated protein) kinase is a shared target of adenosine A_2A_ receptor agonists and CQ/HCQ for autophagy inhibition in neutrophils exposed to antiphospholipid antibodies (83). NETosis inhibition decreases tissue factor exposure by NETs and activated neutrophils to injured endothelium, thus alleviating the risk of thrombosis initiation (84). Given that CQ/HCQ inhibit IL-1β and TNFα production, thus decreasing neutrophil activation status and the generation of tissue factor-decorated NETs (85), it can be speculated that such convergent mechanisms of CQ/HCQ action might be beneficial during the inflammatory phase of SARS-CoV-2 infection.

Finally, low doses of CQ decreased aggregation of human neutrophils stimulated with the bacterial peptide fMLP (formyl-methionyl-leucyl-phenylalanine), while high doses reduced arachidonic acid liberation and thromboxane B2 formation (86), similar to experiments with stimulated platelets (56).

### Lymphocytes

CQ/HCQ effects on the adaptive arm of the immune response are usually ascribed to de-acidification of endosomes and reduced activation of antigen-presenting cells, impairing antigen processing, and presentation to T cells (see above). However, CQ also suppresses lymphocyte proliferation through inhibition of IL-2 mRNA expression, IL-2 production, and IL-2 responsiveness (34). In addition, CQ/HCQ inhibits Ca^2+^ mobilization, resulting in impaired NFAT (Nuclear factor of activated T-cells) activation and CD69 upregulation (35). Similar to macrophages, CQ affects T cell metabolism, including inhibition of autophagy, mitochondrial respiration, and anaerobic glycolysis, accompanied by decreased cytokine secretion in all T helper (Th) cell subsets, mainly Th1 and Th17 (17, 37, 87). T cells preincubated with CQ for 7 days were fully responsive to a secondary stimulation, suggesting that CQ effects on proliferation were reversible (37). Accordingly, CQ/HCQ administration was beneficial in clinical settings and immunopathological mouse models involving Th1 or Th17 polarization, such as ulcerative colitis or SLE (87, 88).

### Natural Killer (NK) Cells/Innate Lymphoid Cells

Most, if not all, studies involving innate lymphocytes and CQ/HCQ have been conducted on NK cells. Initial *in vitro* reports showed that CQ interfered at a similar extent with cytolytic activities of NK and cytotoxic lymphocytes, suggesting that CQ inhibited common pathways (89). Inhibition of NK cytolytic activities did not alter binding to target cells (90). It is suspected that CQ-induced alkalinization blocks perforin processing and maturation in secretory lysosomes, and thus perforin-dependent cytotoxicity (91). Consequently, in patients with RA, CQ therapy decreases spontaneous and IFN-induced NK activities and ameliorates the disease (92).

In conclusion, CQ/HCQ exerts direct effects on immune cell functions. Interference with lysosomal function, autophagy and signaling results in a large array of immunoregulatory activities, including less efficient immune cell activation and selective inhibition of proinflammatory cytokine production. Fine tuning of these pleiomorphic mechanisms might depend on the tissue and cellular microenvironment, the underlying chronic or acute condition, and finally, on the timing, dosing, and duration of CQ/HCQ administration *in vivo*.

## Effects of CQ/HCQ on Non-Immunological Cell Types

### Lung

CQ/HCQ act on immune cells residing in the airway and pulmonary environments, but also affect structural elements such as epithelium, endothelium, smooth muscle, and fibroblasts, potentially protecting lung tissue from overt damage. CQ inhibits TLR-3 sensing and thus abrogates IL22Ra1 (Interleukin 22 Receptor Subunit Alpha-1) induction in normal human bronchial epithelial cells (28). IL-22 supports cell proliferation and inhibits apoptosis, thus favoring lung epithelial repair under mild viral attack, but retaining a deleterious potential in cases of serious lung viral infections. As demonstrated in A549 cells, a cell line of adenocarcinomic human alveolar basal epithelial cells, activation of TLR-3 by the H1N1 influenza virus induces type I IFN which directly upregulates the expression of IL-22Ra1 in a STAT1-dependent manner (28).

In rat bleomycin-induced pulmonary fibrosis, a model of human idiopathic pulmonary fibrosis, systemic delivery of cholesterol-HCQ nanocarriers attenuated the pathophysiological hallmarks of the disease: lung fibroblast proliferation, lung inflammation, and lung fibrosis (6). At the cellular level, HCQ delivery inhibited NF-κB and extracellular signal-regulated kinases (ERK) 1/2 phosphorylation, subsequent proinflammatory cytokine (TNFα) and profibrotic connective tissue growth factor production, bronchial recruitment of neutrophils and lymphocytes, while increasing macrophage numbers in bronchial lavage fluid.

CQ-induced inhibition of autophagy may prevent or potentiate lung function deterioration, according to the underlying condition, the immune orientation, and associated drug administration. In a murine model of allergic asthma, intranasal prophylactic and therapeutic CQ administration resulted in decreased inflammation and attenuated airway remodeling (93). In a rat model, CQ-induced inhibition of autophagy prevented the development of pulmonary arterial hypertension (51). Conversely, CQ administration following cecal puncture and ligation was associated with more severe acute lung injury and reduced survival at 7 days (52). However, improved survival was observed in murine BALB/c endotoxic shock and attributed to the anti-NLRP3 inflammasome action of CQ, *via* caspase-1 inhibition (73).

Treatment of the human cancer line A549 with CQ and paclitaxel, but not with CQ alone, resulted in autophagy inhibition and mitochondrial dysregulation, accumulation of intracellular reactive oxygen species, induction of apoptosis, and cell cycle arrest (46). Autophagy inhibition by itself does not trigger apoptosis in proliferating cells (94). Inhibition of the autophagic elimination of damaged mitochondria (mitophagy) results in impaired cellular metabolism, but also prevents the release of mitochondria-associated molecular patterns, which act as proinflammatory danger signals (95), together with uncontrolled reactive oxygen species production.

HCQ treatment is beneficial in some patients suffering from surfactant-related lung diseases known as idiopathic interstitial pneumonia and including idiopathic pulmonary fibrosis (66–68), through suspected interference with intracellular processing of surfactant proteins (96).

Finally, as a broad-spectrum agonist of TAS2R, CQ increases the intracellular concentration of Ca^2+^ in airway smooth muscle cells through PLCβ (Phospholipase Lipase C β) activation, inositol-trisphosphate (IP3) production and binding to endoplasmic reticulum IP3 receptor, followed by the mobilization of Ca^2+^ stores (53). As a result, CQ administration results in airway smooth muscle relaxation, even in precontracted airways, thus reversing airway obstruction in human, murine, and guinea pig models (97).

### Endothelium

Cardiovascular effects of CQ/HCQ have been intensively studied in the context of autoimmune diseases. In SLE patients, HCQ treatment is associated with a decrease in circulating endothelin-1, improving endothelial function and counteracting TNFα proinflammatory stimulation in experimental murine and human endothelial HUVEC (Human Umbilical Vein Endothelial Cell) models (18, 40, 49). In addition, through its interference with platelet phospholipase A2, HCQ inhibits platelet aggregation. This inhibition is weak, but, similar to other cationic amphiphilic drugs, HCQ inhibits the production of arachidonic acid from platelet membrane after both thrombin and Ca^2+^ ionophore stimulation (58). Apoptosis of endothelial cells results in increased endothelial permeability, which is associated with underlying tissue injury, notably acute lung injury. An *in vitro* model of human microvascular cells coupled with a C57BL/6J mouse model of ischemia/reperfusion demonstrated that CQ inhibition of autophagy, and thus of caspase-3 anti-apoptotic effect, in a context of ischemia-reperfusion, accentuated lung injury (71). In the latter model, CQ not only exerted deleterious effects *per se*, but also prevented the effects of a beneficial anti-integrin monoclonal antibody.

CQ/HCQ effects differ between normal and diseased cardiovascular systems. In a murine model, CQ inhibited endothelial-dependent coronary vascular relaxation in control mice but increased it in diabetic TALLYHO/Jng mice (97), underscoring the special link between CQ/HCQ and type 2 diabetes which will be detailed below.

Although CQ/HCQ is effective in several inflammatory conditions, inhibition of autophagy may exert deleterious side effects and promote tissue damage and organ failure. For example, CQ/HCQ interference with autophagy and mitophagy disrupts mitochondrial turnover, resulting in the accumulation of damaged mitochondria and increased ROS, leading to impaired kidney function (98). Similar findings were observed in mouse models of acute kidney injury in which CQ/HCQ was shown to worsen the disease (99, 100). This effect is not restricted to the kidney, as CQ may also aggravate heart (50), lung (52), and liver (101) injury and may be potentiated by TAS2R-mediated increase of ROS production.

Taken together, these examples show that the balance of CQ/HCQ anti-inflammatory and anti-autophagic effects at the tissue level is a double-edged sword which might be beneficial or deleterious.

## CQ/HCQ and Conditions Associated with Increased Severity During COVID-19

### Obesity

Overweight and obesity are associated with more severe forms of COVID-19 (69, 102). Chronic low-level inflammation is present in these conditions, and immune responses are altered in both systemic and adipose tissue-resident immune cells (103). Moreover, experimental data in mouse models revealed systemic immune dysregulation with decreased T cell progenitors in bone marrow and thymus, thymus involution, and less efficient production of antigen-specific CD8 T cells, reminiscent of clinical observations of decreased influenza vaccine protection in obese subjects (103). In healthy normal weight subjects, the resident immune population of adipose tissue is Th2-skewed and placed under the control of the anti-inflammatory adipokine adiponectin, secreted by adipocytes. A switch from adiponectin to leptin results in a repolarized local environment with increased levels of proinflammatory cytokines and chemokines such as TNFα, IL-6, and MCP-1/CCL2, which in turn drive inflammatory phenotypes of immune cells (104). Overproduction of TNFα in the adipose tissue of obese individuals chronically stimulates lipolysis and impairs triglyceride storage. It is well-known that CQ reduces lipolysis and ameliorates systemic lipid metabolism (105, 106). CQ-induced reduction in lipolysis depends on adipose tissue macrophages through the alteration of lysosome function (107). Autophagy inhibition and PPARγ2 (Peroxisome Proliferator-Activated Receptor γ2) proteosomal degradation are also observed with systemic administration of CQ, leading to impaired adipogenic differentiation and finally blocking high-fat diet-induced obesity (108).

### Diabetes

HCQ is approved as a treatment for diabetes and CQ/HCQ administration to diabetic patients with COVID-19 might be associated with better outcomes (69). However, mechanistic studies suggest that CQ/HCQ may exert opposing effects in different organs, e.g., heart, kidney, or vasculature, according to the type of diabetes. For example, CQ decreased markers of kidney injury: apoptosis, oxidative stress, mitochondrial dysfunction and TGFβ1 (Transforming growth factor β1) expression in human renal proximal tubular cells exposed to high glucose levels, and albuminuria and histopathologic disruption of tubular epithelium and caliber in a mouse model of streptozotocin-induced (type 1) diabetes (39). Conversely, CQ contributed to cardiac function deterioration in mice with diabetic cardiomyopathy. CQ-induced inhibition of autophagy led to divergent mechanistic effects in type 1 and type 2 diabetes models (109). At baseline, autophagy was increased in type 1 diabetic cardiomyopathy through insulin resistance-related intracellular deficiency of glucose (“starvation”), but was inhibited in type 2 diabetes, as a consequence of a cellular overload of nutrients. Consequently, CQ affected more adversely type 2 diabetic cardiomyopathy (109).

## Mechanisms of Direct and Indirect Antiviral Action of CQ/HCQ

CQ was one of four molecules identified among a library of 384 FDA-approved drugs as *in vitro* inhibitors of human coronaviruses replication (110). The three other drugs were chlorpromazine, loperamide, and lopinavir. CQ displayed broad spectrum effects against HCoV (human coronavirus)-229E (an alphacoronavirus), MERS-CoV (Middle East Respiratory Syndrome Coronavirus, lineage C of betacoronaviruses), and SARS-CoV (lineage B of betacoronaviruses, which includes SARS-CoV-2). *In vitro* experiments in Vero and Huh 7 cells confirmed that direct antiviral effects of CQ were exerted predominantly at early steps of viral replication of MERS-CoV. CQ adjunction to Vero or Huh 7 cell cultures 1 h prior to MERS-CoV infection resulted in almost complete inhibition of viral replication. In contrast, CQ adjunction 1 h after the infection did not alter viral replication; however, the ability to reverse the cytopathic effect persisted (110). CQ/HCQ antiviral effect on SARS-CoV-2 *in vitro* was documented shortly after the outbreak initiation, as a potent inhibition of viral replication in pre-and post-infection experiments in Vero E6 cells, with virions stalled in early endosomes and prevented from reaching the late endosome/lysosome compartment (111–113). Post-infection results suggest that CQ also interferes with later steps of viral replication, downstream of viral binding and entry.

CQ/HCQ deacidification of intravesicular pH results in virions carrying abnormally glycosylated envelope proteins, which are less infective and less prone to induce a strong cytopathogenic effect (2, 4, 29).

Overall, viral entry, fusion with endosomes and exit might be the critically affected steps.

## CQ/HCQ and COVID-19: Pros and Cons in the Context of Immunomodulatory Therapeutics

**Figure 3:**
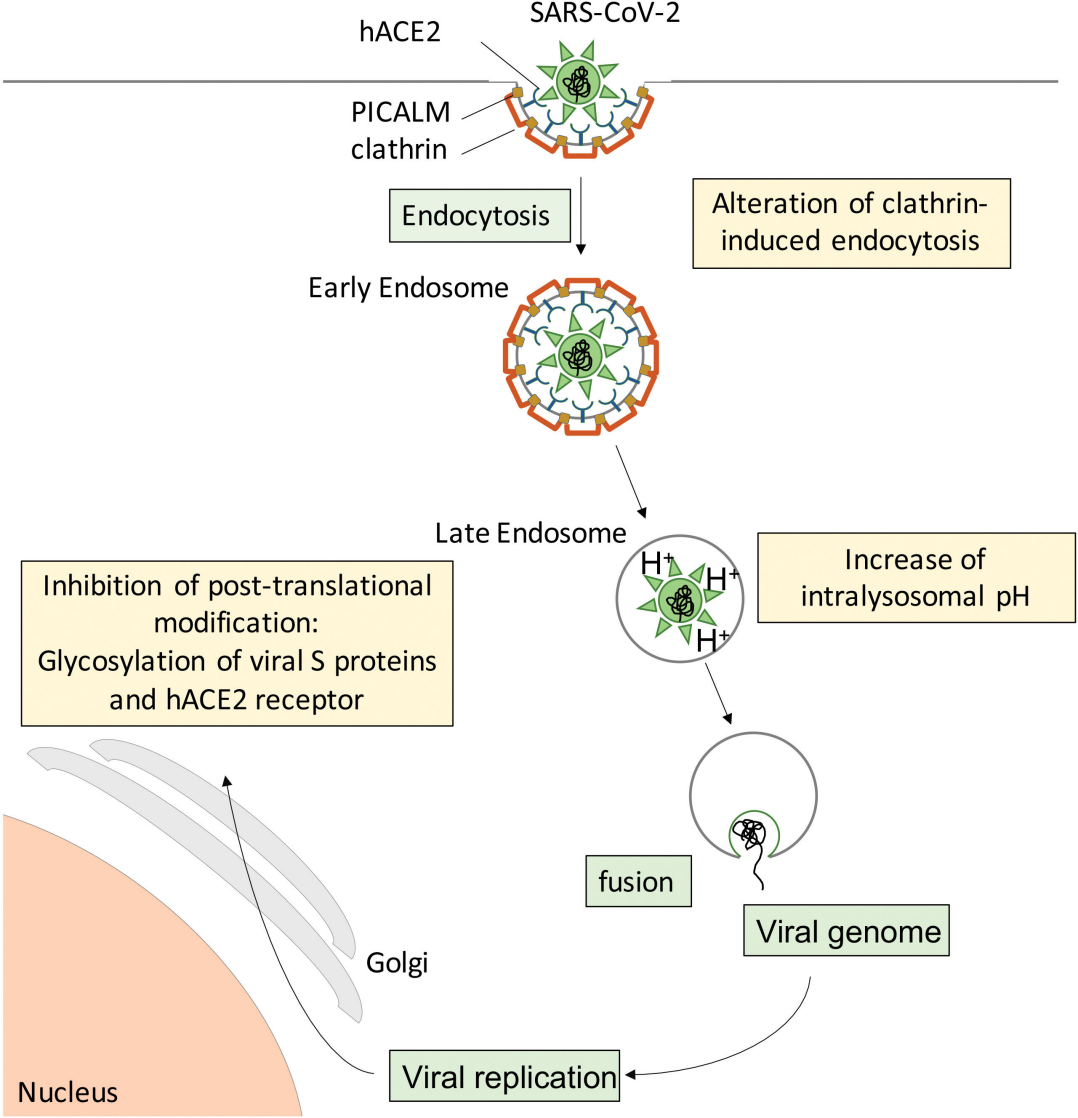
Suspected cellular mechanisms of CQ/HCQ direct anti-SARS-CoV-2 activity. Spike S proteins from SARS-CoV-2 interact with hACE2 at the surface of target cells to induce clathrin-mediated endocytosis. CQ/HCQ may interfere with viral endocytosis by downregulating both PICALM and clathrin expression. In addition, CQ accumulation in the endolysosomal compartments increases the intravacuolar pH and reduces the fusion between the viral envelope and the endosomal membrane, resulting in reduced viral genome translocation in the cytoplasm and thus reduced viral replication. In addition, CQ/HCQ interfere with pH-dependent glycosyltransferase activity of critical enzymes in the Golgi apparatus, thus preventing proper glycosylation of viral S proteins and their host receptor hACE2 and leading to decreased infectivity of the virions.

During the preparation of this manuscript, the first reports on CQ/HCQ effects in COVID-19 patients were published (3, 111, 114–116). However, there was extreme variability in study design, study population, time after symptom onset, sample size, CQ/HCQ dosage and duration of treatment, concurrent administration of other molecules, while the methodology often did not meet the usual standards (117), leaving the reader without clear, comparable data. From a pathophysiological viewpoint, studies endorsing beneficial effects of CQ/HCQ treatment report rescued lung function, mitigated clinical and radiological abnormalities, and shortened duration of virus presence in nasopharyngeal and body fluid samples, allowing earlier discharge from hospital and reducing the risk for further spread of SARS-CoV-2 in the population. These findings suggest that CQ/HCQ administration to COVID-19 patients, especially at an early stage of the disease, provides a “flatten the curve” effect. This could start with a less aggressive viral attack on the host (Figure 3), suboptimal receptor binding, delayed delivery to the endosomes, impaired proteolysis and release of viral RNA in the cytoplasm, while exerting protective effects on lung and vascular systems, providing regulated and repolarized immune responses blunted for hyperactivation and finally a valuable gain of time for the immune system to mount an efficient immune response including adaptive features.

Combined therapeutic schemes associating CQ/HCQ and other molecules could be more efficient in fighting COVID-19, e.g., CQ and remdesivir (111), or HCQ and azithromycin (114). Remdesivir is an adenosine analog which impairs viral RNA synthesis, resulting in broad-spectrum antiviral effects (111). Conversely, azithromycin is a macrolide widely used against airway infections, including prophylactic schemes for chronic obstructive bronchopulmonary diseases (118). Perhaps more importantly for the management of COVID-19, azithromycin is endowed with a bimodal action on the immune system. Azithromycin administration at the early stage of a bacterial infection would promote host defenses, while at later stages with ongoing inflammation this molecule displays an array of immunomodulatory effects spanning epithelial cell protection, airway smooth muscle relaxation, inhibition of fibroblast proliferation, downregulation of neutrophil oxidative and chemotactic responses, extensive macrophage (including alveolar macrophage) modulation with M1 to M2 repolarization and inhibition of TNFα, GM-CSF (Granulocyte-macrophage colony-stimulating factor) and several proinflammatory cytokine production, and finally dendritic cell maturation toward a regulatory phenotype (119). Moreover, azithromycin accumulates in lysosomes, and HCQ has been proposed as a chemosensitizer for improved biodisponibility and prolonged efficacy of lysosomotropic drugs (7). The synergy between HCQ and azithromycin supports the emerging concept of treating SARS-CoV-2 infections with immunomodulators/immunosuppressants (119, 120), at least until a specific antiviral treatment is available.

*In vitro* and clinical data suggest that CQ/HCQ could be useful as a means of preventative control of type I IFN-induced immune activation in SARS-CoV-2 patients (116, 121), although other randomized, controlled studies questioned these findings (122, 123). Recent reports suggest that CQ/HCQ may not act as a rescue medication for severe cases or advanced forms with multiorgan failure (124). Experimental data show that pre-existent IL-1 induced hyperactivation of macrophages counteracts CQ/HCQ effects (47). Similarly, *in vitro* experiments demonstrated the loss of CQ/HCQ antiviral effects when added to cell cultures after multiple rounds of viral replication (2). Critical immune conditions and a “cytokine storm” are associated with severe forms of SARS-CoV-2 infection (120, 125). Such “cytokine storms” are a hallmark of severe forms of other viral infections, including dengue, SARS-CoV and MERS-CoV (17, 126). The “cytokine storm” step might be beyond the reach of the immune modulation and non-specific antiviral properties of CQ/HCQ. Experimental evidence suggests that COVID-19-related cytokine profile is the result of immunosuppression and high inflammatory responses, rather than a classical “cytokine storm” (127). These findings support the view that CQ/HCQ optimal administration should be initiated early during the natural history of COVID-19 (128), rather than at a time point when an uncontrollable inflammatory response, hypoxia, respiratory distress, and multiple organ failure are unfolding in a severe condition. In such uncontrolled cases, ongoing randomized controlled studies involving biotherapies that target the C5a/C5aR pathway [eculizumab (129); avdoralimab NCT04371367] or IL-6/IL-6R pathways [tocilizumab (130)] are promising.

## Conclusion: From *In Vitro* Hopes to Clinical Efficiency

Conceptually, the use of antimalarial drugs as antiviral therapeutics relies on a combination of direct antiviral and indirect anti-inflammatory and immunomodulatory properties. Similarly, the severity of viral infections results from a combination of direct and indirect mechanisms. Among the former, uncontrolled viral replication and cytopathogenic effect are the most prominent, while the latter consist of an inappropriate immune response, with massive release of cytokines and mediators from infected target cells and activated immune cells. The monocyte-macrophage axis, helper and cytotoxic T cells, and innate lymphoid cells are at the stage front of this reaction, although much is yet to be learnt about the complex immune response inside infected tissues and at the hemopoietic level. The pleiomorphic immune responses reported in COVID-19, together with the impreparation of most healthcare systems to a novel pandemic agent at least partially explain the ongoing debate on CQ/HCQ utility, indications, and dosage. The ongoing clinical trials should provide the missing data for defining beyond any reasonable doubt the place of CQ/HCQ in the management of SARS-CoV-2 infection.

## Author Contributions

MM, AB, and AD conducted the literature search. JV and BD extracted, synthesized the data, and wrote the manuscript. JV, BD, and SM created the tables and figures. J-LM provided critical comments on the manuscript. All authors contributed to the conception of the review, interpretation of the data, and the revision of the manuscript.

## Conflict of Interest

JV reports personal fees from Thermo Fisher Scientific, personal fees from Meda Pharma (Mylan), personal fees from Beckman Coulter, personal fees from Sanofi, outside the submitted work. The remaining authors declare that the research was conducted in the absence of any commercial or financial relationships that could be construed as a potential conflict of interest.
